# A continent-wide effort and solidarity at curbing COVID-19 pandemic: the Africa task force for novel coronavirus (AFTCOR) infection prevention and control technical working group’s experience

**DOI:** 10.1186/s12889-023-15706-8

**Published:** 2023-05-15

**Authors:** Elijah Paintsil, Yewande Alimi, Mohammed Abdulaziz, Onyema Ogbuagu, Folasade Ogunsola, Suzan Joseph Kessy, Emilio Horsney, Christopher Lee, Karen Brundney, Tochi Okwor, Patrick Kabwe, Ariyo Waheed, Anna Vondran, Radjabu Bigirimana, Olayinka Ilesanmi, Diana Nambatya Nsubuga, Tajudeen Raji, Wessam Mankoula, Chikwe Ihekweazu, John Nkengasong

**Affiliations:** 1 Department of Pediatrics, Yale School of Medicine, New Haven, CT USA; 2 Africa Centres for Disease Control and Prevention, Addis Ababa, Ethiopia; 3 Department of Medicine, Yale School of Medicine, New Haven, CT USA; 4 Department of Medical Microbiology and Parasitology, College of Medicine, University of Lagos, Lagos, Nigeria; 5 UK Public Health Rapid Support Team, Public Health England, London, UK; 6 Resolve to Save Lives, New York City, USA; 7 Columbia University Irving Medical Center, New York City, USA; 8 Nigeria Centre for Disease Control, Abuja, Nigeria; 9 Infection Control African Network, Cape Town, South Africa; 10 Eastern Africa Reproductive Health Network (EARHN), Nairobi, Kenya

**Keywords:** Centers for Disease Control and Prevention, Coronavirus disease, COVID-19, Infection Prevention and Control, Disease outbreaks, Pandemics

## Abstract

A continent-wide Africa Task Force for Coronavirus with its six technical working groups was formed to prepare adequately and respond to the novel Coronavirus disease (COVID-19) outbreak in Africa. This research in practice article aimed to describe how the infection prevention and control (IPC) technical working group (TWG) supported Africa Centre for Disease Control and Prevention (Africa CDC) in preparedness and response to COVID-19 on the continent. To effectively address the multifaceted IPC TWG mandate of organizing training and implementing rigorous IPC measures at healthcare service delivery points, the working group was sub-divided into four sub-groups—Guidelines, Training, Research, and Logistics. The action framework was used to describe the experiences of each subgroup. The guidelines subgroup developed 14 guidance documents and two advisories; all of which were published in English. In addition, five of these documents were translated and published in Arabic, while three others were translated and published in French and Portuguese. Challenges faced in the guidelines subgroup included the primary development of the Africa CDC website in English, and the need to revise previously issued guidelines. The training subgroup engaged the Infection Control Africa Network as technical experts to carry out in-person training of IPC focal persons and port health personnel across the African continent. Challenges faced included the difficulty in conducting face-to-face IPC training and onsite technical support due to the lockdown. The research subgroup developed an interactive COVID-19 Research Tracker on the Africa CDC website and conducted a context-based operation and implementation research. The lack of understanding of Africa CDC’s capacity to lead her own research was the major challenge faced by the research subgroup. The logistics subgroup assisted African Union (AU) member states to identify their IPC supply needs through capacity building for IPC quantification. A notable challenge faced by the logistics subgroup was the initial lack of experts on IPC logistics and quantifications, which was later addressed by the recruitment of professionals. In conclusion, IPC cannot be built overnight nor can it be promoted abruptly during outbreaks of diseases. Thus, the Africa CDC should build strong national IPC programmes and support such programmes with trained and competent professionals.

**Supplementary Information:**

The online version contains supplementary material available at 10.1186/s12889-023-15706-8.

## Background

Over the last four decades, Africa has experienced several infectious disease outbreaks including human immunodeficiency virus, Ebola virus, and the recent Severe Acute Respiratory Syndrome Coronavirus-2 (SARS-CoV-2) [1–4]. These disease outbreaks and efforts to respond to them continue to reveal the inadequate pandemic preparedness and the fragile state of Africa’s health systems [5]. These characteristics, therefore, highlight the need for a cohesive, collaborative network of public and private partners working across borders and around the world to prevent, detect, and respond effectively to outbreaks of both endemic and emerging infectious diseases.

Despite efforts by governments in Africa and their development partners, there are still major gaps in the implementation of the full tenets of the 2005 International Health Regulations, a treaty that aims to prevent, protect against, control and provide a public health response to the international spread of disease [6]. Most countries in Africa continue to fall short of achieving the core capacities of the International Health Regulations [7]. Moreover, African countries are burdened with inadequate public health infrastructure and regulatory systems, and research expertise that can delay evidence-based decision-making [8].

Given the above mentioned challenges and the perenial threats of infectious diseases outbreaks, Heads of States of the African Union (AU) and the leadership of the AU Commission launched the Africa Centres for Disease Control and Prevention (Africa CDC) on January 31, 2017 [10]. The establishment of the Africa CDC ushered in a new public health order for Africa’s health security and a renewed commitment to accelerate the implementation of the 2005 International Health Regulations [9, 10]. Africa CDC was tasked to work with member states in partnership with the World Health Organization (WHO) and other development partners to strengthen member states’ capacity in four strategic priority areas within five years: (1) expand health-related surveillance and innovative information systems; (2) establish functional clinical and public health laboratory networks in the five geographic subregions of Africa; (3) support member states’ public health emergency preparedness and response plans; and (4) strengthen public health science for improved decision making and practice [11]. On the third anniversary of Africa CDC, the teething organization encountered its most critical litmus test—the declaration of SARS-CoV-2 as a pandemic with some cases reported on the continent [12].

As Africa braced for the epidemic, the continent activated many strategies to prevent the importation of any suspected or confirmed COVID-19 case on the continent. Shortly afterward, the first case of COVID-19 on the continent was reported from Cairo on February 14, 2020, by the Egyptian Ministry of Health and Population [13]. The second COVID-19 case on the continent was reported on February 27, 2020, in an individual who had travelled from Italy to Lagos, Nigeria [14]. On March 11, 2020, the WHO declared the COVID-19 outbreak a global pandemic and therefore the need for countries to take mitigating actions to control the virus [15]. Thus, the threat of the virus to Africa in the face of already fragile health systems on the continent was real and needed urgent attention [16].

A continent-wide strategy was adopted for preparedness and response to COVID-19. On February 22, 2020, the AU Commission, Africa CDC, and WHO convened an emergency meeting in Addis Ababa, Ethiopia, of all ministers of health of Africa’s 55 member states to commit to act fast and collectively develop and implement a strategy [17]. At the meeting, the Africa Task Force for Coronavirus (AFTCOR) was formed with the mandate of coordinating and implementing the adopted continent-wide strategy [17]. The membership of AFTCOR is multisectoral including representations from AU member states, governmental agencies, critical non-governmental stakeholders, development partners, academic institutions, and healthcare workers from different countries [17]. To effectively coordinate response efforts across the continent, six technical working groups were set up under AFTCOR: (1) Infection prevention and control (IPC), (2) Surveillance, (3) Clinical management of persons with severe COVID-19 infection, (4) Laboratory diagnosis and subtyping, (5) Risk communications, and (6) Supply chain and stockpiling medical commodities.

This research in practice article aimed to describe how the IPC TWG supported Africa CDC in preparedness and response to COVID-19 on the continent. The main objectives were to: (1) apply action research in the activities of the IPC TWG; (2) describe the contribution of the IPC TWG towards the continent-wide strategy in breaking the chain of COVID-19 transmission; and (3) share the lessons learnt during this iterative and ongoing process.

## Methods and processes

### Formation and composition of IPC TWG

The IPC TWG was established in February 2020 with the following objectives: to support the implementation of rigorous IPC measures at healthcare service delivery points; to provide IPC training for national IPC focal persons, healthcare workers, and port health personnel; to provide on-site technical assistance to member states for the development and implementation of protocols in their healthcare facilities; and to develop and update comprehensible, practical and context specific IPC guidance based on evolving scientific evidence around the prevention, pathogenesis. and transmission of SARS-CoV-2.

Furthermore, the IPC TWG supports member states to ensure that there are minimum standards for safety and IPC in healthcare facilities as relates to water, sanitation, hygiene, ventilation, and adequate supply and availability of personal protective equipment (PPE). The membership of the IPC TWG is multisectoral and interprofessional drawing from AU member states, multilateral partners, Regional Economic Communities, the private sector, donors, foundations, and other experts from different countries within and beyond the African continent. Table 1 shows the membership and composition of IPC TWG with their expertise and affiliations.

**Table 1:** Infection Prevention and Control Technical Working Group membership

Name	Title	Organization
Amadou Cheick Tidiane CISSE	Liason Officer	Africa Centre for Disease Control and Prevention (Africa CDC)
Amy Elizabeth Barrera-Cancedda	Infection Prevention and Control (IPC) Programme Consultant	Resolve to Save Lives
Anna Vorndran	Executive Manager, Infection Control African Network (ICAN)	ICAN, South Africa
Anthony Twyman	Training Coordinator, ICAN	ICAN
Buyiswa Lizzie M (Nee Sithole)	Training Coordinator, ICAN	ICAN
Christopher T. Lee	Director, Global Epidemic Preparedness and Response Team, Resolve to Save Lives	Resolve to Save Lives (RTSL)
Dan Dano Ibrahim	Head of Quality Service / Project Manager	Africa Union Development Agency- New Partnerships for Africa’s NEPAD (AUDA-NEPAD)
Diana Nambatya Nsubuga	Regional Coordinator the Eastern Africa Reproductive Health Network,	Eastern Africa Reproductive Health Network (EARHN)
Elijah Paintsil	Director, Pediatric Infectious Diseases Fellowship Training Program, Yale School of Medicine	Yale University
Emilio Hornsey	Senior Infection Prevention and Control Nurse UK Public Health Rapid Support Team	Public Health England
Eric Beohou	Public Health consultant	AUDA-NEPAD
Ezinne Onwuekwe	Public Health Fellow	Africa CDC
Folasade Ogunsola	Chairperson, Infection Control African Network (ICAN)	Infection Control Africa Network, Nigeria /University of Lagos
Giorgia Gon	Assistant Professor	London School of Hygiene and Tropical Medicine
Hanna Dantata	Public Health Consultant	AUDA-NEPAD
Hiwot Moges	Public Health Fellow	Africa CDC
Idowu Lateef	Public Health Fellow	Africa CDC
Ifeoluwapo Elizabeth Obasanya	IPC Technical Assistant	Nigeria Centre for Disease Prevention and Control
Joseph Mthetwa	Project Management Officer for the United Nations Economic Commission for Africa	AUDA-NEPAD
Karen Brudney	Senior Expert Adviser to the Centers for Disease Control Division of HIV and Tuberculosis	Columbia University Irving Medical Center
Kelly O. Elimian	Prevention, Programmes and Knowledge Management Officer	Nigeria Centre for Disease Prevention and Control
Koné Datolo	Public health consultant	AUDA-NEPAD
Mamadou Saliou Kaalifa Diallo	Independent Consultant, M&E Progrogrammes	AUDA-NEPAD
Marshall Thomas	COVID-19 Response, Learning and Preparedness	Resolve to Save Lives
Maureen Moyo-Chilufya	Researcher	AUDA-NEPAD
Minja Frank	Outreach Program Director. Yale Radiology Global Outreach Program	Yale University
Mohammed Abdulaziz	Head of Division, Disease Control and Prevention | Ag. Head of Division, Surveillance	Africa CDC
Muhammad Shakir Balogun	Field Epidemiology Training Programme Resident Advisor, Nigeria	African Field Epidemiology Network
Onyema Ogbuagu	Director of the Yale AIDS Program HIV clinical trials program	Yale University
Olayinka Stephen Ilesanmi	Event Based Epi-Analyst	Africa CDC
Patrick Nguku	Field Epidemiology Training Programme Resident Coordinator, Nigeria	African Field Epidemiology Network
Patrick Kabwe	Event Based Epi-Analyst	Africa CDC
Paul Tanui	Public health consultant	AUDA-NEPAD
Radjabu Bigirimana	Program Lead, African Volunteer Health Corps (AVoHC)	Africa CDC
Tajudeen Raji	Head of Public Health Institutes and Research	Africa CDC
Suzan Joseph Kessy	Public Health Fellow	Africa CDC
Thaddee Niyoyitungira	Public Health Officer	Africa CDC
Tigistu Adamu	Associate Medical Director, Jhpiego	Johns Hopkins Program for International education in Gynaecology and Obstetrics
Tochi Okwor	Director Prevention, Programmes and Knowledge Management/ Antimicrobial Resistance (AMR) programme Coordinator	Nigeria Centre for Disease Prevention and Control
Waheed Ariyo Bakare	Public Health Officer / Southern Africa Regional IPC Focal Point	Africa CDC
Woldemedhin T. Haile	Senior public health consultant	AUDA-NEPAD
Yewande Alimi	AMR and One Health programme Coordinator Africa CDC	Africa CDC

These members were recruited and engaged with consideration of their expertise in IPC and public health in general; subsequently, they were provided with terms of reference for the work. For efficiency and to effectively address the multifaceted IPC TWG mandate, the working group was sub-divided into 4 sub-groups—Guidelines, Training, Research, and Logistics.

### Action research framework

**Fig. 1:**
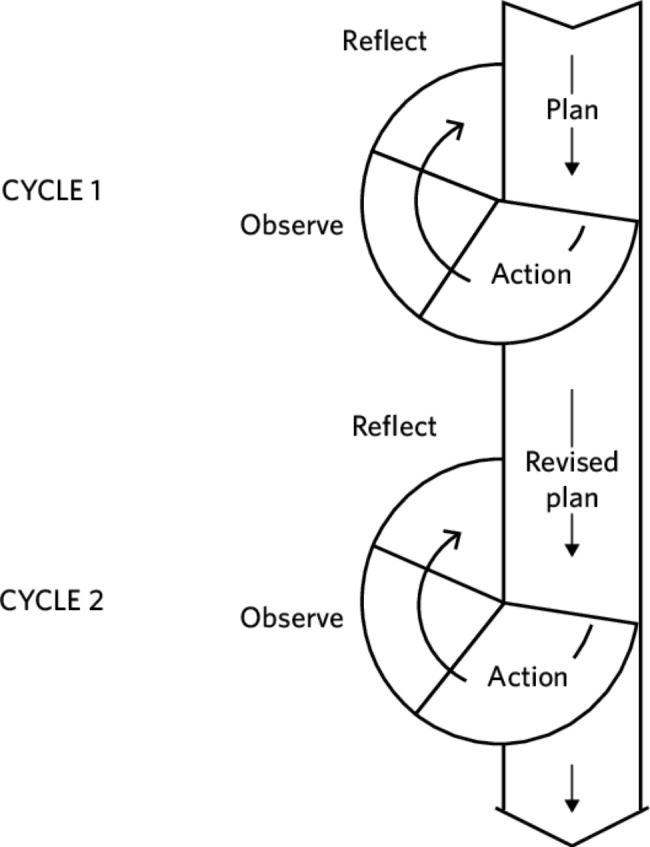
Kemmis and McTaggart’s action research spiral model

The IPC TWG started its work at a time when the science and data on the transmission dynamics of COVID-19 were evolving at a fast pace. Therefore, we adopted the action research framework that allows for an experiential learning approach to continuously refine the methods, data, and interpretation in light of knowledge and experience gained from earlier activities and evolving COVID-19 data. Action research, also known as ‘participatory action research’ or ‘action science and action learning, is an approach commonly used for improving conditions and practices in healthcare environments [18]. In using the action research framework, we based our activities on Kemmis and McTaggart’s action research spiral model [19] (Fig. 1).

This model comprises a spiral of reflective cycles of planning, acting, observing, and reflecting. These stages overlap with in-built allowance for redundancy such that initial plans could become obsolete but lessons learned from them, providing a template for future activities in the context of a fluid COVID-19 pandemic.

Each of the four sub-groups held a weekly meeting. These meetings were the main discussion and brainstorming fora and served as a decision-making platform for the sub-groups. Where necessary, members presented a review of literature on topics assigned to them, developed survey forms, and discussed and approved concept papers and guidance documents. Progress was noted on an Action Plan Tracker in a shared google document which was accessible to all members. In addition, on a weekly basis, the entire IPC TWG met to discuss subgroup activities, and as a group to plan, act and observe, reflect and re-plan. The entire AFTCOR group (comprised of the six technical working groups) met weekly to track the overall state of the pandemic, successes, and challenges of AFTCOR activities, and engage with external partners. During these weekly meetings, the IPC TWG provided updates on its activities and these presentations informed AFTCOR strategies to address implementation challenges on the continent.

### Collaborations and partnerships

As the adage goes in Africa, ‘It takes a village to raise a child’; the work of the IPC TWG would be impossible without collaborations and partnerships with several organizations, agencies, and institutions. The African Union is a continental union that promotes the unity and solidarity of the 55 member states. Through her public health agency, the Africa CDC, AU was able to coordinate and integrate partners and collaborating agencies to strengthen the continet’s response to the COVID-19 outbreak. A Consortium of technical and organizational experts from organizations in Africa and United States was formed to strengthen the COVID-19 outbreak response in Africa. These were made up of Health Maxima, Infection Control Africa Network (ICAN), Johns Hopkins Program for International education in Gynaecology and Obstetrics; London School of Hygiene & Tropical Medicine, Public Health England; Resolve to Save Lives; United States Centers for Disease Control and Prevention; United Kingdom Public Health Rapid Support Team (UK-PHRST), and Yale University.

AFENET is a not-for-profit networking and service alliance of Field Epidemiology and Laboratory Training Programs present in more than 31 countries. AFENET served as the vendor, contractor, and administrator when Africa CDC was established, and thus had a lot of professional knowledge of administration needed on the Consortium. WHO led global disease prevention and control efforts. The expertise of the WHO was needed to have a robust COVID-19 response team.

## Results and outputs

In this research in practice article, we report the IPC TWG activities and outputs from March 2020 to December 2020. This section is presented according to the activities, successes, and challenges of the four IPC TWG sub-groups—Guidelines, Training, Research, and Logistics.

### Guidelines sub-group

The IPC guidelines subgroup was established in March 2020 to respond to AU member states’ requests and translate global normative guidance to the African setting. The subgroup provided member states quick access to context-specific and context-appropriate IPC guidance in line with international guidelines from WHO, US Centers for Disease Control and Prevention, and European Centres for Disease Control and Prevention. The guidelines subgroup held weekly meetings between March to October 2020, and then switched to biweekly meetings. Technical experts from participating organizations reviewed all guidance materials before submission to the Africa CDC science group for review and clearance.

Overall, 14 guidance documents and two advisories were drafted, 14 were cleared and published in English. In addition, five of these documents were translated and published in Arabic, while three others were translated and published in French and Portuguese the period under review (Table 2).

**Table 2:** Infection Prevention and Control Technical Working Group Guidance Documents

Guidance Document	Date published	Languages available
Community use of face masks	21 April 2020	English
Personal Protective Equipment for Different Clinical Settings and Activities	30 May 2020	English Arabic
Guidance on Environmental decontamination in the context of COVID-19	30 June 2020	English French Portuguese Arabic
Strategies for managing acute shortages of personal protective equipment during COVID-19 pandemic	29 July 2020	English French Portuguese Arabic
Best Practices for COVID-19 in Primary Healthcare Facilities	15 Aug 2020	English
Advisory on disinfecion cards	30 Nov 2020	English
IPC guidelines for ambulances transferring known or suspected COVID-19 cases	01 Nov 2020	English
Standard operating procedures for caregivers in COVID-19 treatment centres	01 Nov 2020	English
COVID-19 Infection Prevention and Control: Your Questions Answered	01 Sep 2020	English
Guidance on Infection Prevention among Contact Tracers in Africa	14 Aug 2020	English
Guidance on Setting Up an Isolation Ward for COVID-19 Cases	14 Aug 2020	English Arabic
Hand washing facility options for resource limited settings	25 Jun 2020	English Portuguese Arabic
Promoting mask-wearing : A policymaker’s guide	23 Nov 2020	English French
Advisory on Respiratory Protective Equipment	21 Nov 2020	English

The development of guidelines was prioritized by the subgroup using prioritization criteria and upon consensus of the subgroup; the criteria included (i) member state request for guidance or advisory note, (ii) absence of existing global guidance, and (iii) need for translation to African-specific context. In addition to developing IPC guidance for clinical settings, the subgroup also drafted guidlines to limit COVID-19 transmission in community settings including recreational facilities, airports, schools, and the effective use of non-medical masks. The subgroup responded to member State’s specific needs; one of such was the development of a COVID-19 mask guidance document for policymakers highlighting instances of successful implementation from across the continent to support Africa mask week in November 2020.

These guidelines (7–11) were easily adopted by member states, reducing the time gap between the development of IPC guidelines and implementation. Guidelines were published on the Africa CDC website and social media pages shared via the AFCTOR mailing list to member states and direct distribution of hard copies during each country’s IPC training. The Africa CDC website is primarily in English, thus, there was minimal uptake from non-Anglophone countries. Therefore, guidelines were published in Arabic, French, and Portuguese. Also, due to the rapidly evolving nature of the COVID-19 pandemic with new evidence emerging frequently, the guidelines subgroup had to review and revise previously issued guidelines to reflect contemporaneous evolving scientific evidence on SARS-CoV-2 transmission modes and epidemiology to keep them up to date.

### Training sub-group

The training subgroup commenced activities in February 2020 before the index case in Africa. This was in recognition of and response to the insufficiency of human and material IPC resources on the continent. The mandate of the training subgroup was to lead the development of competency-building activities focused on preparedness, response, and recovery from COVID-19 and other infectious disease outbreaks. The specific objectives were to: (1) conduct COVID-19 IPC training-of-trainers for member states’ national IPC focal persons and port health IPC personnel; (2) support member states with sub-national capacity building in IPC; and (3) improve the knowledge and competences of IPC focal persons, frontline healthcare workers in healthcare facilities and the community to prevent COVID-19 transmission during healthcare delivery.

**Fig. 2:**
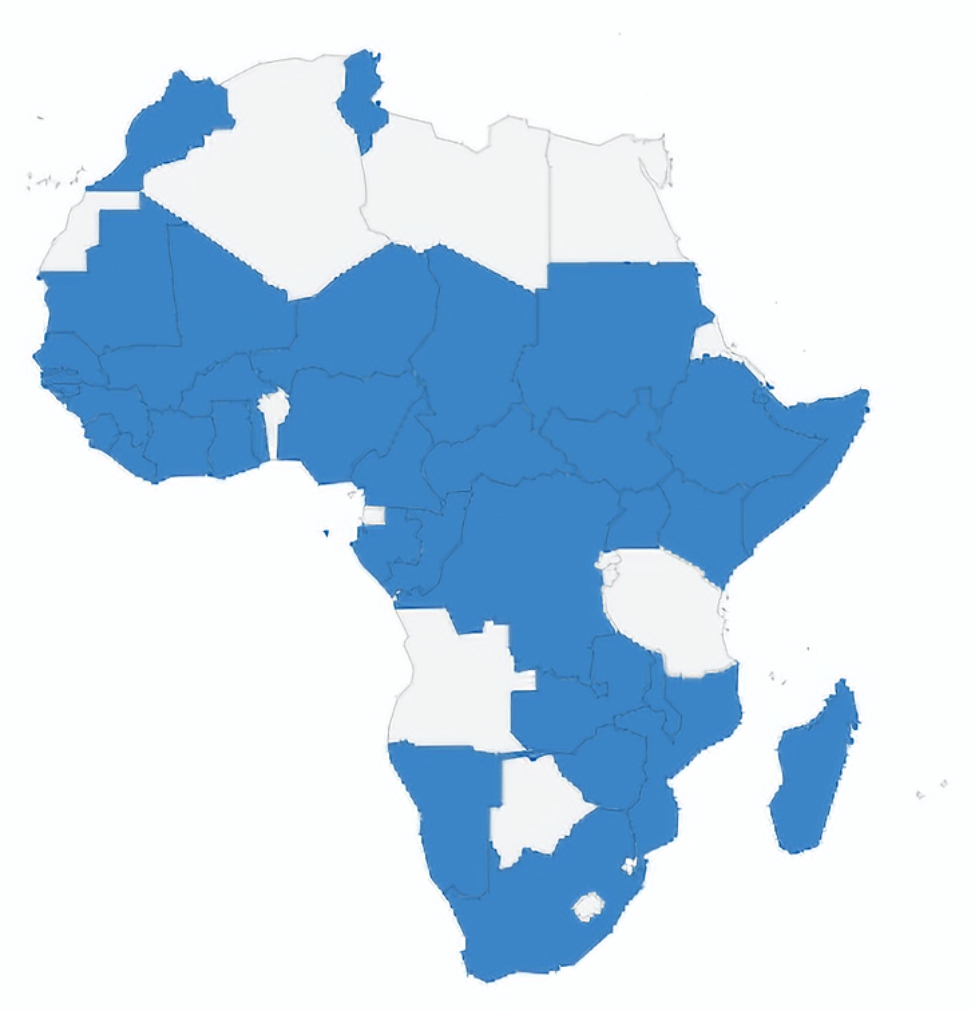
Overview of in-country capacity building of infection prevention and control training in member countries, May 2020-December 2021

The first activity involved the engagement of ICAN as technical experts to carry out in-person training of IPC focal persons and port health personnel across the African continent as part of the preparedness activities. The goal was to build the capacity of the IPC focal persons to rapidly identify and prioritize the risk of acquiring SARS-CoV-2 during healthcare delivery and apply solutions that minimize, control, or eliminate these risks. ICAN developed the curriculum and worked with the Africa CDC, the Nigeria Centre for Disease Control, WHO African region, and AFENET to provide training. The training was conducted in Abuja, Nigeria, between February 21- 22 and February 24-25-2020 with 23 countries in attendance. (Figure S1) A 3-day training was thereafter conducted in Abidjan, Ivory Coast, from March 11-13, 2020 with the participation of 17 countries. Overall, a total of 80 participants from 36 countries were trained (Fig. 2). The overview of attendance of infection prevention and control training in member countries, May 2020-December 2021 is in supplementary Table S1. The overview of in-country capacity building of infection prevention and control training in member countries, May 2020-December 2021 can be found in supplementary Table S2.

The trainings were conducted in English and French using didactic and practical methods including role play, case studies, and group work. Participants were provided with a sample training pack to facilitate step-down training in their home countries. As a result, step-down training have been carried out in 12 countries with over 647 participants trained between May 2020-December 2021.

Because member state imposed travel restrictions and lockdowns in response to the increasing number of countries who were reporting COVID-19 cases as of the end of March 2020, face-to-face IPC training and onsite technical support became challenging to conduct. Therefore, the subgroup developed a training framework using a blended learning method of technology-mediated learning via webinars and a community of practice for knowledge management practices.

In total, the subgroup reported participation from 50 AU Countries and 53 non-AU countries in the webinar sessions. However, there was an absence or low participation from some countries on the continent. Angola, Libya, Cabo Verde, Equatorial Guinea, and Eritrea were not represented on either the English or French webinars. There was no participation from seven countries in the English webinars (Chad, Central African Republic, Angola, Libya, Cabo Verde, Equatorial Guinea, and Eritrea). Among the 18 Francophone countries, Cabo Verde, Equatorial Guinea, and Seychelles were not represented in the French webinars. During the first wave, many healthcare workers were overwhelmed and could not attend many of the sessions. Figure S2 shows English webinar participants by location. Figure S3 shows French webinar participants by location. Figure S4 shows all participants by location.

The group has run four (4) series of six (6) weekly webinar sessions, each run concurrently in English and French. From April-December 2020, participants from 48 AU countries were trained in English webinar sessions and participants from 37 AU countries were trained in French webinar sessions. Supplementary Table S3 shows the overall number of registrants per series. Figure S5 shows the overview of weekly number of registrants per series. To increase their coverage and accessibility, all webinars were recorded and uploaded on ZOOM cloud and YouTube, with a total of 3,616 views as of 31st December 2020. In addition, the subgroup adopted the use of social media platforms like Facebook. The sessions were streamed live on Facebook and had a total of 5,630 live viewers; 2,539 for the English webinar and 3,091 for the French Webinar. Figure S6 shows the number of peak live viewers per webinar.

To strengthen the support available to healthcare workers and to support IPC implementation in their facilities, a community of practice was set up on Telegram. A recurring theme in the feedback from participants was the clarity, practicality, and contextualization of the content with direct relevance to the African healthcare situation (See Supplementary File 1). Below are direct quotes from participants:

*“This is an excellent presentation which is quite relevant to African health care facilities”* (Participant A).

*“The session was so clear about the African setup of lifestyle”*(Participant B).

*“I was glad to hear more tailored approaches taking into account the resource challenges faced on the ground and understanding the more critical linkages between health, poverty, education, etc. that inform the challenges and solutions for implementing IPC in these countries”* (Participant C).

Participants also found the webinar series and community of practice useful for networking. They suggested that some of the webinars should be targeted at community health workers. In general, the participants asked that the platform should be adopted by the Africa CDC for regular IPC training.

*“I want Africa CDC to keep providing this kind of IPC training for strengthening health system”*(Participant B)

*“I’m excited to join every week. This is needed. The content is reliable and useful in practice. Well done and I hope you will continue till I retire in a few years”*(Participant D)

Other feedback on the webinars organized are found in the supplementary file.

To ensure sustained interest and participation, the subgroup began to issue certificates of participation as an incentive. Challenges experienced in the attendance of the webinars included disruptions with internet connections across several countries as well as differences in time zones. It was also recognized that IPC is practical and while webinars are valuable for knowledge transfer, they are limited in the ability to ensure skills acquisition. Therefore, in the future, the group will explore producing videos and visuals for a demonstration to supplement knowledge acquired through the webinars.

### Research sub-group

The research subgroup was formed and started meeting in April 2020. The IPC TWG leadership acknowledged that in previous outbreaks, when efforts were focused on response, sometimes the opportunity for learning critical lessons and documenting best practices was lost. Cognizant of the need to strengthen IPC research capacity on the continent, the research subgroup was integrated into the TWG structure at the outset.

The mandate of the research subgroup was 3-fold: (1) keeping abreast with IPC-related research; (2) assessing IPC research needs on the continent; and (3) engaging in operation and implementation of scientific research for evidence to inform decision making. To avoid duplication of research conducted by other organizations, the subgroup created databases of research being conducted on the continent to identify evidence gaps and set priorities. We developed an interactive COVID-19 Research Tracker on the Africa CDC website that made navigation of IPC and COVID-19 research more accessible [20].

Although there is a wealth of IPC research activity from high-income settings, we found a paucity of original research data on IPC from Africa and, therefore, the need to translate or adapt high-income research findings to the African setting and context. Some issues were specific to the African experience that did not appear to be addressed elsewhere. Hence, the subgroup developed a working document of identified research needs and focus areas. It also contributed to a COVID-19-related Research and Development Priorities policy paper for COVID-19 in Africa led by the Science Standards and Regulations TWG [21].

Members of the subgroup also conducted operation and implementation research based on identified needs, and in collaboration with each other or external networks. Proposals for priority research areas were submitted to seek intramural Africa CDC funding and extramural funding from other partners. Members of the subgroup were part of several successful applications for grants that are allowing us to meet some of the research needs on the continent. At the time of writing, these studies were at various stages of progress and a brief summary has been included (Table 3).

**Table 3:** COVID-19 related research scope for Africa

Study title	Objectives	Institution	Stage	Funder
Training a continent: a process evaluation of a virtual training on infection prevention and control in Africa in the context of COVID-19	The aim is to evaluate the design, delivery and target audience response to two Infection Prevention and Control (IPC) training activities, and make recommendations for ongoing/ future programming.	London School of Hygiene and Tropical Medicine, University of Lagos	Completed paper submitted	None
Transmission risk of respiratory viruses in natural and mechanical ventilation environments: implications for SARS-CoV-2 transmission in Africa	To conduct a narrative review of evidence on ventilation systems, with emphasis on health structures and settings in Africa.	Yale School of Medicine	Completed and published, paper available at : https://pubmed.ncbi.nlm.nih.gov/32863269/#affiliation-4	None
World Health Organization international case control multicentre study – Assessment of risk factors for COVID-19 in health workers	(i) To characterize and assess the IPC risk factors for SARS-CoV-2 infection in health workers with exposure to COVID-19 patients (ii) To describe the range of clinical presentations for SARS-CoV-2 infection in health workers, including the duration and severity of the disease	Nigerian Centre for Disease Prevention and Control; World Health Organization Headquarters	Study ongoing	World Health Organization
Development and evaluation of resources to support IPC engagement with caregivers in hospitals.	To describe the role and function of family care givers in a hospital setting. To develop tools to support engagement with infection prevention and control and care givers. To pilot delivery of an engagement programme with care givers. To conduct a process evaluation of the pilot intervention	Cameroon Baptist Convention Health Services, London School of Hygiene and Tropical Medicine	Study ongoing	DHSC through the UK-PHRST
COVID-19 droplet protection using face shields: development of methods to measure the effectiveness of face shields for local production and adoption in low-resource settings	To acquire data on why people choose face shields (sometimes called visors), which are being used extensively during the COVID-19 pandemic, especially in low- and middle-income countries, and on how they are worn, to inform how face shields can be best designed to minimize transmission of COVID-19 or other influenza-like illness	University of East Anglia, University of Lagos	Study ongoing	World Health Organization
Electrolysed water for hospital cleaning: pilot study in Nigeria	To test the effectiveness of electrolysed water on hospital surfaces compared to standard products through a pilot laboratory study and a cluster randomized control trial in Nigeria. Pilot objectives: • To assess the feasibility of product application • To select high-touch surfaces • To adapt the procedures for outcome measurement • To estimate the baseline microbiological cleanliness for standard cleaning products • To assess the potential effectiveness of hypochlorous acid • To refine the procedures for allocation concealment	Nigeria Centre for Disease Prevention and Control, London School of Hygiene and Tropical Medicine	Study ongoing	World Health Organization
Infection Prevention and Control Technical Working Group Guidelines Evaluation	Ongoing review of publication utilization. Using an iterative process to inform Guidelines subgroup and refine ongoing activities. Analysis of website data and surveys of key stakeholders, including national IPC focal points and participants in step down training.	Africa Centre for Disease Prevention and Control	Completed: Internal report presentation shared.	None

At the inception of the subgroup, there was limited evidence on the mode of transmission of SARS-CoV-2. Generally, respiratory viruses can be transmitted through contact, droplet, and airborne routes. Most resource-limited healthcare settings lack complex air handling systems to filter air and create pressure gradients that are necessary for minimizing viral transmission. Also, COVID-19 disproportionately affected people living in low socioeconomic conditions [22] who tend to live in more densely populated areas with poor ventilation. Interestingly, little is known about the contribution of environmental factors such as natural or mechanical ventilation in the transmission of SARS-CoV-2. Could environmental factors such as ventilation systems contribute to the high transmissibility of SARS-CoV-2? The subgroup conducted a systematic review of the literature to explore the association between ventilation and the transmission of respiratory viruses like SAR-CoV-2 [23]. We found that when used appropriately, both natural and mechanical ventilation can decrease the concentration of viral aerosols thereby reducing transmission. Although mechanical ventilation systems are more efficient, installation and maintenance costs limit their use in resource-limited settings, while the prevailing climate conditions make natural ventilation less desirable. Cost-effective hybrid systems of natural and mechanical ventilation may overcome these limitations [23, 24].

The subgroup collaborated with the training subgroup to evaluate the virtual IPC webinars. The webinar series were developed incredibly swiftly, to reach as wide an audience as possible, as early as possible in the pandemic. It was felt that this work should be evaluated as robustly as possible as this mode of delivery may inform future activities of the Africa CDC. The scope of the evaluation was agreed upon through discussion with the training organizers and advisory members in a design workshop. Ethical approval was sought and gained from the University of Lagos, Nigeria, and the London School of Hygiene and Tropical Medicine, and the evaluation was conducted between July 2020 and January 2021. A mixed-methods approach was used; with prospective and retrospective data collection included due to the rapid start of training activities. The key findings were that the rapid development of this training was efficient and responsive. The training had reasonable coverage, with over 6000 viewers during the study period, but the numbers varied largely by location. Participants engaged particularly well with live question and answer sessions during the webinars. The African focus of the webinars was appreciated by many participants and the more practical and context-specific, the better it was appreciated. It was challenging to assess the degree to which participants’ level of knowledge or skills improved, or whether the webinars led to actual improvements in participants’ local context. Despite these limitations, the results do support the organization of virtual training (as part of a broader package of measures) as a huge opportunity to improve IPC across the African continent.

Many primary care services including immunization, family planning, HIV and Tuberculosis treatment, and antenatal care were suspended because of concerns related to the potential spread of SARS-CoV-2 in these settings. This was likely to have increased the indirect morbidity and mortality associated with SARS-CoV-2, probably outweighing the direct effects of the virus itself. As a result, the subgroup prioritized research using surveillance data from member states to analyze the impact of COVID-19 in special populations—children and adolescents, and people living with HIV (PLWH). These two projects are underway. On one hand, the study of COVID-19 in children and adolescents seeks to describe the epidemiology of COVID-19 and risk factors associated with a severe infection in children and adolescents in Africa, with the following specific aims: (1) To describe the clinical features of COVID-19 infection in children and adolescents aged 0–19 years in Africa; and (2) To identify factors associated with the severe disease among children and adolescents with COVID-19 infection in Africa. On the other hand, the study of COVID-19 in PLWH sought to: (1) assess whether PLWH in Africa are at increased risk of acquiring COVID-19 and/or experiencing severe COVID-19 compared to those without HIV; (2) assess predictors including outcomes including COVID-19 acquisition and COVID-19 severity (outpatient and inpatient setting); (3) assess what comorbidities among PLWH are associated with worse outcomes (including mortality) with COVID-19; (3) describe demographic and clinical characteristics of PLWH with COVID-19 in Africa; and (4) assess the predictors of COVID-19 severity and mortality (including intensive care unit stay, invasive mechanical ventilation and /or death) among PLWH in Africa.

The research subgroup has been an excellent platform to encourage debate, share information and focus on team members’ research activities. The subgroup has built relationships that have led to the development of successful proposals. It has also maintained regular contact with the wider Science, Standards, and Regulations TWG in Africa CDC which was undergoing a period of evolution and development at the same time. This collaboration is essential to ensure that our activity is aligned with the strategic objectives of AFTCOR.

We acknowledge many limitations to the work of the research subgroup. At the time of the group’s inception, members did not yet understand the capacity of Africa CDC to lead their research. This process evolved during the response and is still developing. Many of the group members were volunteering in their own time and had several external pressures to balance. The make-up of the group evolved, and new members brought energy and additional perspectives.

### Logistics sub-group

The logistics subgroup had the mandate to assist member states with identifying their IPC supply needs through capacity building for IPC quantification, and regulatory agencies within the continent that provide appropriate standards and regulations for IPC materials and their production, as well as provide information on where supplies could be sourced. Due to some intersection in mandates, the IPC logistics subgroup frequently interacted with supply chain TWG to align activities and promote synergy. The pandemic presented a unique logistics challenge for the continent, and as a global shortage of PPE was envisaged, there was a need to set up a logistics subgroup of the IPC TWG to strategise on ways of ensuring the availability of PPE and other IPC commodities to healthcare workers across the continent. Moreover, the pandemic highlighted the fragility of international supply chains and the dependency of many African countries on imported PPE. There was thus an urgent need to identify and map the continent-based manufacturers of PPE to encourage member states to optimally utilize locally produced PPE and make adequate plans for stockouts by developing the domestic capacity to locally manufacture these commodities.

At its inception, the subgroup held meetings weekly. During periods where some tasks needed more attention, meetings were held more frequently, and decisions to hold these meetings were based on consensus. Collaboration with various experts in the logistics subgroup provided the needed resource persons to produce high quality deliverables.

The limitations to the role of the logistics subgroup was an initial lack of experts on IPC logistics and quantification, however, this was boosted by the recruitment and participation of epidemiology consultants supported by the Africa Union Development Agency- New Partnerships for Africa’s NEPAD (AUDA-NEPAD). Another limitation was the inability of Africa CDC to host and maintain online databases and maps that require real-time updates. This capacity is still in development.

One of the key achievements of the logistics subgroup was the creation of an interactive map of local PPE manufacturers on the continent. This exercise exposed/highlighted the gaps in the local production of PPE on the continent and informed the continent-wide workshop that was organized to promote the local production of PPE in AU member states (https://africacdc.org/download/medical-ppe-production-in-africa-promoting-local-manufacturers-to-support-the-covid-19-response-workshop-report/). The workshop brought together different stakeholders involved in the production and regulation of PPE on the continent. Furthermore, the identified local PPE manufacturers were linked to the African Medical Supplies platform, a procurement platform accessible only to AU Member states, to further the agenda of providing access to locally produced PPE. The workshop was well received with over 500 participants in attendance. Following the workshop, the logistic subworking group developed a draft document on a strategic approach for sustaining the engagement of PPE manufacturers and meeting future production needs.

Furthermore, informed by a needs assessment of the capacity of member states to generate accurate IPC needs, the logistics subgroup is currently planning for a series of training to build capacity among health care workers and program managers in IPC commodity quantification. To achieve this, an IPC quantification tool was developed. This tool is expected to help member states correctly quantify the IPC needs for various settings. Working closely with AUDA-NEPAD, a developmental agency of the AU, seven epidemiologists who were contracted to help member states with IPC estimates based on epidemiological trends in respective countries assisted with developing the tool and training materials. The subgroup will prioritize future work to focus on training healthcare workers and program managers in the quantification of IPC commodities. The main challenges encountered by the subgroup were the lack of post-market surveillance and limited laboratory testing to determine the quality of PPE being used in Africa. However, efforts are ongoing through the Africa medical devices regulatory harmonization forum and a soon to be set-up Africa Medicines Agency, to develop capacity for both regional and continent level regulatory capacity to support national-level regulatory agencies.

## Reflections and lessons learned

The COVID-19 pandemic (similar to the Severe Acute Respiratory Syndrome and Middle East Respiratory Syndrome and West African Ebola virus disease outbreaks) once again placed IPC in the spotlight, highlighting the impact of existing IPC gaps on healthcare systems, across the continent and globally.

This research in practice manuscript details the work and processes of the AFTCOR IPC TWG’s COVID-19 response and highlights the unique challenges in implementing IPC activities on the African continent. At the onset of the pandemic, the TWG identified challenges such as the lack of guidelines specific to COVID-19, a dearth of trained health professionals and IPC experts, insufficient stockpiles and domestic manufacturing of PPE, underfunding of national IPC programs, and in several countries, the absence of national IPC programs and regulatory capacity.

Despite the lessons learned from previous epidemics with strong evidence that IPC is effective for controlling epidemics, Africa’s response, in general, is often characterized by cycles of crisis response followed by neglect and complacency. Many countries on the continent perceive IPC as a ‘reactive approach to epidemics’ as opposed to a critical component for pandemic preparedness, health systems strengthening, health worker protection, and patients’ safety. Indeed, the COVID-19 global pandemic has shown the importance of building a more resilient healthcare system with effective IPC as a key to avoiding or mitigate outbreak impact.

Regarding IPC materials, Africa CDC utilized various mechanisms to procure and distribute PPE to member states to mitigate the PPE shortage through collaborations with technical partners like the United Nations Children’s Fund and World Food Programme. There remains an urgent need to address the challenges related to the PPE shortage in healthcare facilities, in Africa, by promoting local production of PPE that meet international standards, and countries are encouraged to explore the African Continental Free Trade Agreement as a mechanism to procure PPE manufactured on the continent.

This manuscript is important as it documents the experiences gained during the AFTCOR response; a unique continental collaboration that provides the opportunity to share evidence-based IPC practices with other continents and can inform the future of IPC research on the continent. Africa needs to identify its own IPC research priorities and set up sustainable funding mechanisms to advance independent research capacity on the continent, without relying solely on funding from high income settings, which results in a continued bias in research agenda and authorship [25]. An African-led and focused research agenda will allow the research needs of the continent to be given the attention they deserve [25]. There should also be linkages across African research institutions such as the African Academy of Sciences, African Universities, Africa CDC, WHO regional offices in Africa and other institutions.

The continental response further highlights why Africa must continue to prioritise the safety of healthcare workers and patients. The outcomes of the AFTCOR IPC TWG highlight the impact of coordinating efforts and leveraging partnerships for a multisectoral approach to IPC activities. Continental and regional organizations; Africa CDC, Nigeria Centre for Disease Control, ICAN, WHO (AFRO), and WHO Eastern Mediterranean region are encouraged to maintain the collaborative approach for providing IPC training, developing IPC guidelines and policies such as Africa CDC’s IPC legal framework and WHO’s national strategy for the protection of healthcare workers, aimed at supporting the implementation of international standards and WHO’s core components for IPC at national and healthcare facility levels. The legal framework currently being finalized provides an opportunity for member states to develop IPC standards that will also cover Water, Sanitation, and Hygiene (WASH) standards. Protection of the health workforce also needs to be addressed in a holistic manner that involves both occupational health and IPC experts.

Beyond institutional collaborations, Africa CDC should develop a database of IPC experts on the continent and across the world willing to support continental efforts for implementing IPC. The work of the AFTCOR IPC TWG showcases how global experts can significantly contribute to advancing IPC across the continent, by supporting the development and review of guidelines informed by member states’ needs and context. This mechanism can be explored to support other ongoing epidemics across the continent to ensure the rapid dissemination of evidence-based IPC guidelines.

Improving IPC requires strong political commitment, trained IPC specialists, IPC programs with a dedicated budget, strict adherence to protocols for detection and response, provision of adequate supplies and equipment for patient care, and adherence to hygiene, sanitation, and infectious waste management, and measure compliance with standards. There is a need for domestic financing to improve IPC infrastructure and service delivery as an integrated aspect of routine healthcare delivery which is important to ensure safety in healthcare delivery and readiness to respond to disease outbreaks.

This work further reiterates the urgent need to establish and strengthen IPC programs at the national and facility levels for preparedness and readiness for future pandemics. National governments should prioritise the implementation of IPC activities across all cadres of healthcare workers, in line with the continental position of support by the AU Heads of State and Government, for the long-term implementation of IPC activities.

## Limitations

The Consortium was built based on existing partnership with certain organizations. Thus, there was no formal documentation reflecting the engagement of these organizations. The achievements of the TWG were limited particularly regarding the tracking and reporting on healthcare worker COVID-19 infection in the continent. Going forward, this needs to receive more attention from the TWG including tracking and reporting on rates of COVID-19 vaccination among healthcare workers. There was also not much direct engagement with national IPC focal persons in the continent. The effort to engage these national focal persons was not very successful and perhaps is a reflection of the lack of properly instituted national IPC programmes manned by competent and trained personnel and recommended by the WHO core components for infection prevention and control programme. Going forward, the TWG will prioritise supporting member states to build these IPC programmes and will engage with member states through these focal persons. A forum for meeting and communicating plans and strategies with these IPC focal persons will be created.

## Conclusions

IPC cannot be built overnight nor can it be promoted abruptly during times of crisis such as outbreaks and pandemics and be expected to function optimally. The Africa CDC should position herself strategically in IPC responsibilities including research and development agenda to make sure that the agency can cater to old threats and be prepared for future health emergencies. This can be done through building of strong national IPC programmes and supporting such programmes with trained and competent IPC professionals.

The legal framework currently being finalized provides an opportunity for member states to develop IPC standards that will also cover WASH standards. Protection of the health workforce also needs to be addressed in a holistic manner that involves both occupational health and IPC. Following the workshop on PPE, the logistics subgroup made the following recommendations: Conduct a rapid situational analysis to establish the current situation regarding PPE in each country with a view to establishing a support system for countries with limited capacity in accreditation, regulation, production, and human capacity to address the complex challenges of PPE.

It was further recommended to adopt and widely share guidelines through available in-country channels such as organizations or associations in the health sector. The purpose of such guidelines would be to provide direction on repurposing resources to meet the PPE needs of each country. Where such guidelines do not exist or are inadequate, they should be strengthened to meet the needs as here described. Such guidelines will also regulate the production, procurement, and distribution of PPE, ensure a level playing field that would accommodate small-scale businesses as well as provide checks against monopoly by big business. It was further recommended to set up an online registration for local manufacturers, followed by certification. This would make it easier to maintain standards, and regulate the activities of players, including assigning quotas to manufacturers as needed. The Africa CDC should work with health ministers in AU countries to also identify or source funds to set up a production and training institute as a hub to serve the needs of the continent.

## Electronic supplementary material

Below is the link to the electronic supplementary material.

Supplementary File: **Table S1**: Overview of attendance of infection prevention and control training in member countries, May 2020-December 2021. **Table S2**: Overview of in-country capacity building of infection prevention and control training in member countries, May 2020-December 2021. **Figure S1**: IPC in country Capacity building(21-25 February 2020. 36 countries were trained, and 80 participants were trained). **Figure S2**: English Webinar Participants by Location. **48 AU Countries are represented** in the webinars. Among the 7 countries, not represented include Chad, Central African Republic, Angola, Libya, Cabo Verde, Equatorial Guinea, Eritrea. In attendance, 48 non-AU countries were represented. **Figure S3**: French Webinar Participants by Location. **37 AU Countries are represented** in the English webinars. Among the 18 Francophone countries, 03 countries were not represented include, Cabo Verde, Equatorial Guinea, and Seychelles. In attendance, 15 non-AU countries were represented. **Figure S4**: All Webinar Participants by Location. 50 AU Countries are represented in the webinars. Among the 5 countries, not represented include Angola, Libya, Cabo Verde, Equatorial Guinea, Eritrea. In attendance, 53 non-AU countries were represented. **Table S3**: Overall number of registrants per series. **Figure S5**: Overview of weekly number of registrants per series. **Figure S6**: Number of peak live viewers per webinar. Recorded webinars are uploaded on zoom cloud and You Tube and distributed for a total of 3,616 views.

## Data Availability

All data generated or analysed during this study are included in this published article [and its supplementary information files].

## List of abbreviations

Africa CDC: Africa Centre for Disease Control and Prevention
AFENET: African Field Epidemiology Network
AFTCOR: Africa Task Force for Novel Coronavirus AU:African Union
AUDA-NEPAD: Africa Union Development Agency- New Partnerships for Africa’s NEPAD
PLWH: People Living With HIV
PPE: Personal Protective Equipment
IPC: Infection Prevention and Control
TWG: Technical Working Group
WASH: Water, Sanitation, and Hygiene
WHO: World Health Organization

## References

[1] World Health Organization. HIV/AIDS. https://www.afro.who.int/health-topics/hivaids#. Accessed 21 April 2022.

[2] World Health Organization. Ebola virus disease. https://www.who.int/health-topics/ebola#tab=tab_1. Accessed 19 September 2021.

[3] World Health Organization. Coronavirus (COVID-19). https://www.afro.who.int/health-topics/coronavirus-covid-19. Accessed 20 January 2022.

[4] Africa Centers for Disease Control and Prevention. Coronavirus Disease 2019 (COVID-19). https://africacdc.org/covid-19/. Accessed 30 March 2022.

[5] World Health Organization. The State of Health in the WHO African Region: An analysis of the status of health, health services, and health systems in the context of the Sustainable Development Goals. https://www.afro.who.int/. Accessed 16 January 2021.

[6] World Health Organization. Implementation of the International Health Regulations, the Functioning of the International Health Regulations. (2005): Report of the Review Committee on (2005) in relation to Pandemic (H1N1) 2009. Sixty-fourth World Health Assembly. Provisional agenda item 13.2. A64/10. 2011. https://apps.who.int/gb/ebwha/pdf_files/WHA64/A64_10-en.pdf. Accessed 15 December 2021.

[7] Talisuna A, Yahaya AA, Rajatonirina SC (2019). Joint external evaluation of the International Health Regulation (2005) capacities: current status and lessons learnt in the WHO African region. BMJ Global Health.

[8] Nkengasong JN, Maiyegun O, Moeti M (2017). Establishing the Africa Centres for Disease Control and Prevention: responding to Africa’s health threats. Lancet Global Health.

[9] Nkengasong J, Djoudalbaye B, Maiyegun O (2017). A new public health order for Africa’s health security. Lancet Glob Health.

[10] Africa Centers for Disease Control and Prevention. Our History. https://africacdc.org/about-us/our-history/. Accessed 15 March 2022.

[11] Africa Centers for Disease Control and Prevention. Africa Centres for Disease Control and Prevention launches five-year strategic plan to improve surveillance, emergency response and prevention of infectious diseases. https://africacdc.org/news-item/africa-centres-for-disease-control-and-prevention-launches-five-year-strategic-plan-to-improve-surveillance-emergency-response-and-prevention-of-infectious-diseases/. Accessed 9 January 2022.

[12] Africa Centers for Disease Prevention and Control. Responding to COVID-19 in Africa: Finding the Balance (Part IV) and Calls to action. https://africacdc.org/download/responding-to-covid-19-in-africa-finding-the-balance-part-iv-and-calls-to-action/. Accessed 15 February 2022.

[13] World Health Organization. Update on COVID-19 in the Eastern Mediterranean Region. Feb, 16., 2020. http://www.emro.who.int/media/news/update-on-covid-19-in-the-eastern-mediterranean-region.html. Accessed 10 February 2020.

[14] World Health Organization African region. A second COVID-19 case is confirmed in Africa. https://www.afro.who.int/news/second-covid-19-case-confirmed-africa#. Accessed 15 April 2021.

[15] World Health Organization. WHO Director-General’s opening remarks at the media briefing on COVID-19–11. March 2020. https://www.who.int/director-general/speeches/detail/who-director-general-s-opening-remarks-at-the-media-briefing-on-covid-19---11-march-2020. Accessed 12 June 2021.

[16] Paintsil E. COVID-19 threatens health systems in sub-Saharan Africa: the eye of the crocodile. J Clin Invest 2020. 10.1172/JCI138493 PMC7260015 32224550

[17] Nkengasong JN, Mankoula W (2020). Looming threat of COVID-19 infection in Africa: act collectively, and fast. Lancet.

[18] Lingard L, Albert M, Levinson W (2008). Grounded theory, mixed methods, and action research. BMJ.

[19] Kemmis S, McTaggart R, editors. The Action Research Planner. (ThirdEdition) Waurn Ponds. Deakin University Press; 1988.

[20] African Centres for Disease Prevention and Control. COVID-19 Research Tracker. https://africacdc.org/covid-19/covid-19-research-tracker/. Accessed 28 February 2021.

[21] African Centres for Disease Prevention and Control. Policy Paper: Research and Development Priorities for COVID-19 in Africa. https://africacdc.org/download/policy-paper-research-and-development-priorities-for-covid-19-in-africa/. Accessed 20 December 2021.

[22] Provisional Death Counts for Coronavirus Disease (COVID-19): Weekly Updates by Select Demographic and Geographic characteristics. 2020. https://www.cdc.gov/nchs/nvss/vsrr/covid_weekly/index.htm#ExcessDeaths. Accessed 13 May 2020.

[23] Sopeyin A, Hornsey E, Okwor T et al. Transmission risk of respiratory viruses in natural and mechanical ventilation environments: implications for SARS-CoV-2 transmission in Africa. BMJ Glob Health 2020; 5(8). 10.1136/bmjgh-2020-003522 PMC7462043 32863269

[24] World Health Organization. Natural Ventilation for Infection Control in Health-Care Settings.WHO Publication/Guidelines. 23762969

[25] Kasprowicz VO, Chopera D, Waddilove KD (2020). African-led health research and capacity building- is it working?. BMC Public Health.

