# Supplementary material for: A continent-wide effort and solidarity at curbing COVID-19 pandemic: the Africa task force for novel coronavirus (AFTCOR) infection prevention and control technical working group’s experience
Source: BMC Public Health. 2023 May 15;23:893. doi: 10.1186/s12889-023-15706-8 (PMC10184057; doi:10.1186/s12889-023-15706-8)
Supplement: Supplementary file 1 — Supplementary File: Table S1: Overview of attendance of infection prevention and control training in member countries, May 2020-December 2021. Table S2: Overview of in-country capacity building of infection prevention and control training in member countries, May 2020-December 2021. Figure S1: IPC in country Capacity building(21-25 February 2020. 36 countries were trained, and 80 participants were trained). Figure S2: English Webinar Participants by Location. 48 AU Countries are represented in the webinars. Among the 7 countries, not represented include Chad, Central African Republic, Angola, Libya, Cabo Verde, Equatorial Guinea, Eritrea. In attendance, 48 non-AU countries were represented. Figure S3: French Webinar Participants by Location. 37 AU Countries are represented in the English webinars. Among the 18 Francophone countries, 03 countries were not represented include, Cabo Verde, Equatorial Guinea, and Seychelles. In attendance, 15 non-AU countries were represented. Figure S4: All Webinar Participants by Location. 50 AU Countries are represented in the webinars. Among the 5 countries, not represented include Angola, Libya, Cabo Verde, Equatorial Guinea, Eritrea. In attendance, 53 non-AU countries were represented. Table S3: Overall number of registrants per series. Figure S5: Overview of weekly number of registrants per series. Figure S6: Number of peak live viewers per webinar. Recorded webinars are uploaded on zoom cloud and You Tube and distributed for a total of 3,616 views. [file 12889_2023_15706_MOESM1_ESM.docx]

**Supplementary File**

Table S1: Overview of attendance of infection prevention and control training in member countries, May 2020-December 2021

| **Country** | **Participants** | **Number of trainings** |
| --- | --- | --- |
| Burundi | 30 | 1 |
| Cameroon | 50 | 2 |
| Democratic Republic of Congo | 25 | 1 |
| Ghana | 74 | 3 |
| Kenya | 25 | 1 |
| Mauritius | 50 | 2 |
| Nigeria | 366 | 6 |
| South Africa | 118 | 2 |
| South Sudan | 50 | 2 |
| Zambia | 25 | 1 |
| Zimbabwe | 297 | 3 |
| The Gambia | 25 | 1 |

Table S2: Overview of in-country capacity building of infection prevention and control training in member countries, May 2020-December 2021

| **Country** | **Participants** | **Number of trainings** |
| --- | --- | --- |
| Burundi | 25 | 1 |
| Cameroon | 50 | 2 |
| Democratic Republic of Congo | 25 | 1 |
| Ghana | 50 | 2 |
| Kenya | 25 | 1 |
| Mauritius | 50 | 2 |
| Nigeria | 150 | 6 |
| South Africa | 147 | 6 |
| South Suudan | 50 | 2 |
| Zambia | 25 | 1 |
| Zimbabwe | 25 | 1 |
| Gambia | 25 | 1 |


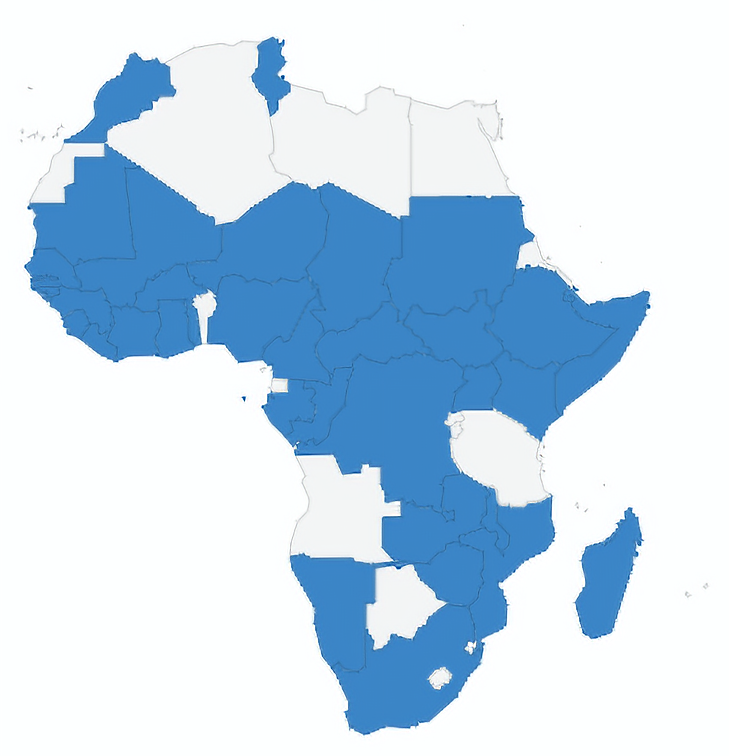


Figure S1**:** IPC in country Capacity building

*(21-25 February 2020. 36 countries were trained, and 80 participants were trained)*


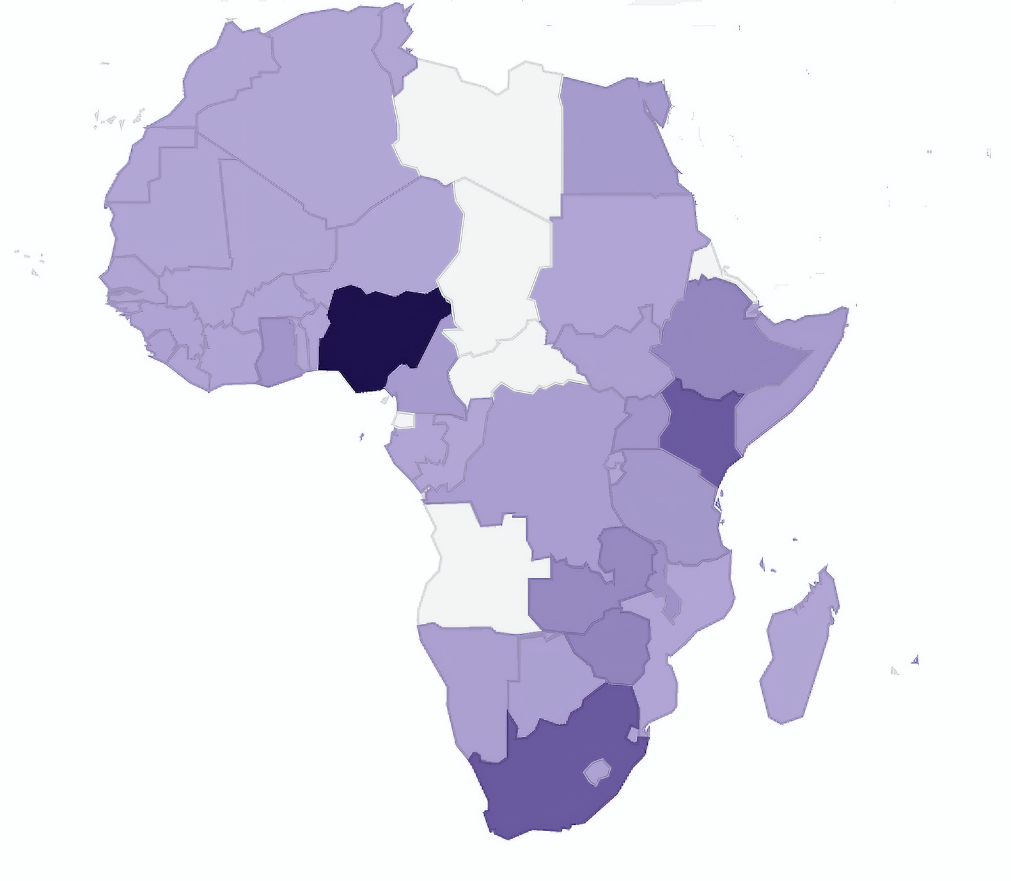


Figure S2: English Webinar Participants by Location

***48 AU Countries are represented*** *in the webinars. Among the 7 countries, not represented include Chad, Central African Republic, Angola, Libya, Cabo Verde, Equatorial Guinea, Eritrea. In attendance, 48 non-AU countries were represented.*


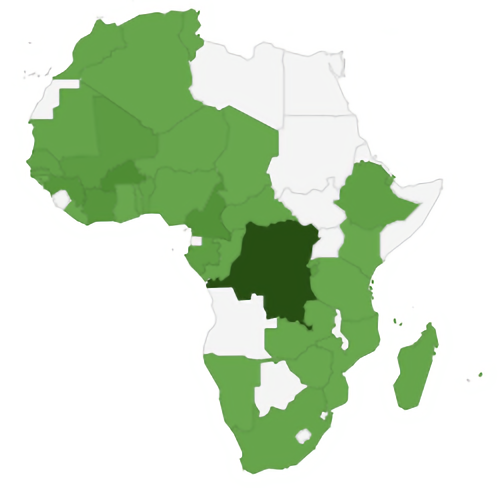


Figure S3: French Webinar Participants by Location.

***37 AU Countries are represented*** *in the English webinars. Among the 18 Francophone countries, 03 countries were not represented include, Cabo Verde, Equatorial Guinea, and Seychelles. In attendance, 15 non-AU countries were represented.*


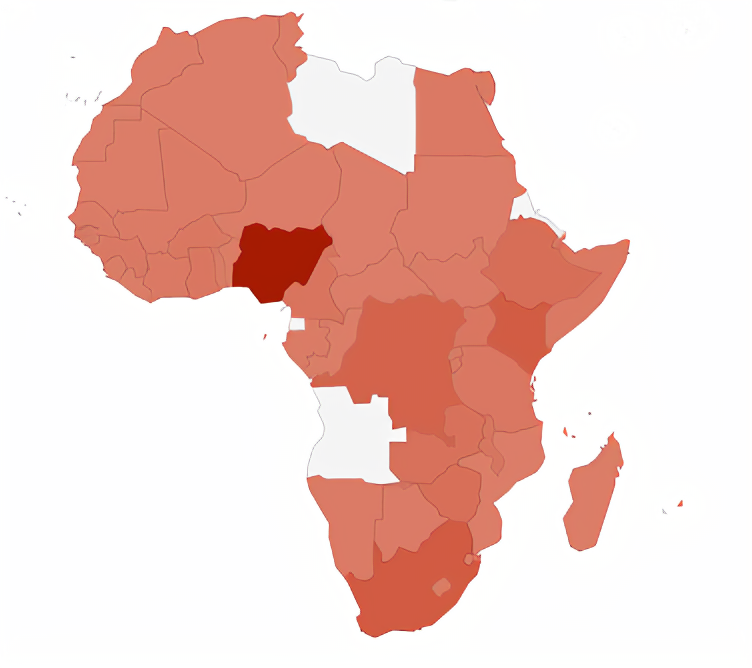


Figure S4: All Webinar Participants by Location

***50 AU Countries are represented*** *in the webinars. Among the 5 countries, not represented include Angola, Libya, Cabo Verde, Equatorial Guinea, Eritrea. In attendance, 53 non-AU countries were represented.*

| **Language** | **Series 1** | **Series 2** | **Series 3** | **Series 4** |
| --- | --- | --- | --- | --- |
| English | 3726 | 2389 | 1705 | 1385 |
| French | 699 | 554 | 747 | 298 |

**Table S3:** Overall number of registrants per series.

**Figure S5:** Overview of weekly number of registrants per series

Figure S6: Number of peak live viewers per webinar

Recorded webinars are uploaded on zoom cloud and You Tube and distributed for a total of 3,616 views

**Feedback on the webinars organized by Africa Centre for Disease Prevention and Control**

- *I was glad to hear more tailored approaches considering the resource challenges faced on the ground and understanding the more critical linkages between health, poverty, education, etc. that inform the challenges and solutions for implementing IPC in these countries* (Participant A)*.*
- *Excellent presentation which is quite relevant to African health care facilities (*Participant B)
- *The session was so clear about the African setup of lifestyle* (Participant G).
- *Was well organized though time extended over* (Participant E)
- *The webinar is a very good tool, and the topic was very good, it, however, was much more inclined toward health facilities and biosecurity related to animal health should be prominent as well* (Participant M)
- *All the topics are useful and serve as an instrument for interrupting the spread of Covid19 infection. we recommend training on disease control in the African region using the same platform* (Participant H)
- *The Africa CDC webinar discussion is important and helps to improve knowledge in my practice as a health practitioner* (Participant T)
- *I find the webinar presentations of great interest and excellent contribution to COVID-19 IPC* (Participant Y)
- *I want Africa CDC to keep providing this kind of IPC training for strengthening the health system* (Participant B).
- *I enjoyed the Webinar in terms of meeting senior professional colleagues/professors, and it also really impacted my knowledge on issues related to COVID -19 and other infectious diseases* (Participant S)
- *I'm excited to join every week. This is really needed. The content is reliable and useful in practice. Well done and I hope you will continue till I retire in a few years (*Participant D).
- *IPC need to be strengthened in all our healthcare facilities, HAIs have always been a problem in African countries, and our surveillance for HAIs is poor, especially in Nigeria. Thank you all for your immense contribution to improving IPC practice* (Participant F)*.*
- *It’s a great initiative to train the HCW regarding IPC practices. A research to assess the change in practices in the facilities from where the participants belong is highly recommended* (Participant C).
- *Very brief, clear, and readable with links to read further* (Participant B)*.*
- *We need to train community volunteers on personal hygiene on COVID-19*

(Participant F).

- *The training was very interesting and useful for us. Since Africa CDC is trying the best to empower with training as well as experience sharing. Thanks a lot* (Participant D).
